# A Multicenter Retrospective Study of 58 Patients With Primary Thyroid Diffuse Large B Cell Lymphoma

**DOI:** 10.3389/fendo.2020.00542

**Published:** 2020-08-28

**Authors:** Jianing Yi, Pingyong Yi, Wei Wang, Huan Wang, Xinyu Wang, Hanjia Luo, Peizhi Fan

**Affiliations:** ^1^Surgical Department of Breast and Thyroid Gland, The First Affiliated Hospital of Hunan Normal University/Hunan Provincial People's Hospital, Changsha, China; ^2^Department of Oncology, Changsha Kexin Cancer Hospital, Changsha, China; ^3^Department of Oncology, Affiliated Cancer Hospital of Xiangya School of Medicine, Central South University, Changsha, China; ^4^Surgical Department of Breast and Thyroid Gland, Xiangya Second Hospital of Central South University, Changsha, China; ^5^Surgical Department, Xiangya Hospital of Central South University, Changsha, China

**Keywords:** thyroid, lymphoma, large B-cell, prognosis, treatment

## Abstract

**Background:** Primary thyroid diffuse large B cell lymphoma (DLBCL) is a rare type of extranodal lymphoma; optimal treatment methods and the key prognostic factors have not been established.

**Methods:** The clinical data of 58 patients with primary thyroid DLBCL from January 2007 to December 2017 were collected. The Kaplan–Meier method and log-rank tests were used for the survival analysis. Cox regression analysis was performed to evaluate the prognostic factors.

**Results:** The follow-up time was 6–120 months; 5-year overall survival (OS) and progression-free survival (PFS) were 73 and 61%, respectively. Single-factor analysis showed that IPI, Ki-67, treatment modalities, Hans classification, Myc/Bcl-2 protein co-expression, and administration of rituximab had a significant effect on the 5-year OS and PFS (*P* < 0.05), while age, sex, Bcl-2 protein expression, Myc protein expression, tumor stage, tumor size, Hashimoto's thyroiditis, and B symptoms were not associated with prognosis (*P* > 0.05). Multivariate risk regression analysis revealed that Myc/Bcl-2 protein co-expression, treatment modalities, and rituximab were independent prognostic factors (*P* < 0.05).

**Conclusions:** Patients with primary thyroid DLBCL who received combination chemotherapy with radiotherapy had a better prognosis. Surgical treatment alone was not associated with the prognosis and is used only for diagnosis. Rituximab could improve the survival time of patients.

## Introduction

Primary thyroid lymphoma (PTL) is an uncommon lymphoma that develops in the thyroid gland, accounting for ~2% of all extranodal lymphomas; its morbidity is higher in elderly females with chronic Hashimoto's thyroiditis (1). Generally, the main complaint is neck swelling; some patients also show B symptoms such as night sweats, fever, and weight loss (2). Histopathologically, DLBCL is the main type of PTL; other pathological subtypes include follicular lymphoma, mucosa-associated lymphoid tissue lymphoma, small lymphocytic lymphoma, and T-cell lymphoma (2, 3). Surgical biopsy or core needle biopsy can be performed to diagnose the disease. Previous studies on primary thyroid DLBCL were done on the basis of limited data due to the rarity of the disease, and there is no consensus on optimal treatment modalities and key prognostic factors. To address these issues, this study aimed to investigate treatment modalities and clinicopathological characteristics correlated with survival in patients with primary thyroid DLBCL, so as to find useful information about treatment option and key prognostic factors.

## Materials and Methods

### Clinical Data

Clinical data were obtained from the medical records of 58 patients with primary thyroid DLBCL, who were diagnosed and treated at Hunan Provincial People's Hospital, Hunan Cancer Hospital, Changsha Kexin Cancer Hospital, Xiangya Hospital, and the Second Xiangya Hospital from January 2007 to December 2017. Age, sex, treatment modality, stage, Hashimoto's thyroiditis existence, LDH (lactate dehydrogenase), IPI (international prognostic index), tumor size, Hans classification, Ki-67 and Bcl-2 protein expression, Myc protein expression, B symptom, and overall survival status were evaluated. Core needle biopsy, surgical biopsy, and surgical excision were used for diagnosis. Patients who had systemic disease were excluded from the evaluation. Follow-up data were calculated from the time of initial surgery on the thyroid gland to the last follow-up. The clinical survey was finished through reviewing patient records, interview, and clinic visits. Ethical approval for this study was obtained from the ethical committee of The First Affiliated Hospital of Hunan Normal University/Hunan Provincial People's Hospital. Written informed consent was obtained from all of the local cohort participants.

### Evaluation Standard

Primary thyroid DLBCL developed in the thyroid gland with or without the involvement of regional lymph nodes and without metastasis to other visceral tissues (1). All samples were reviewed by two pathologists for confirmation of the histological diagnosis. The clinical stages were defined in two categories through computed tomography, ultrasonography, and bone marrow aspiration biopsy: Stage IE refers to lesion localized within the thyroid, and stage IIE refers to lesion confined to the thyroid and regional lymph nodes (3). Fouty percentage was used for cutoff values of for Myc and 70% for Bcl-2 and Ki-67 (4, 5); the cutoff values of other markers assessed in this study were based on previous reports (6).

### Patient Survival

The final follow-up was done on December 1, 2018. Overall survival (OS) was calculated from the time of initial surgery on the thyroid gland to death from any cause, while progression-free survival (PFS) was calculated from the date of diagnosis to the date of initial disease progression, relapse, or death.

### Statistical Analysis

The Kaplan–Meier method was used for the survival analysis and univariate analysis. The Cox regression analysis was performed to evaluate the prognostic factors of patients with primary thyroid DLBCL. The chi-squared (χ^2^) test was performed to compare clinical characteristics, and differences were tested using the two-tailed test. SPSS software (version 21.0, SPSS Inc., Chicago, IL, USA) was used for all statistical analyses. All *p*-values were 2-sided, and a *P* < 0.05 was considered statistically significant.

## Results

### Clinical Characteristics

At the time of diagnosis, of the 58 patients with primary thyroid DLBCL, 43 were women and 15 were men; 15 patients presented bilateral involvement, 21 presented right lesions, and 22 presented left lesions; 18 patients had clinical B symptoms, and 40 had no B symptoms; 28 patients had stage IE lymphoma and 30 patients had stage IIE lymphoma; 31 patients had 0–1 IPI score, and 27 patients had 2–3 IPI score; 24 patients had elevated LDH; and 22 patients had a history of Hashimoto's thyroiditis. The median age of the patients was 65 years (range, 28–78 years), and the average size of the primary thyroid DLBCL was 3.8 cm (range, 1.5–7.6 cm). A total of 30 patients presented involvement of adjacent organs, including 18 patients with neck lymph-node invasion and 12 patients with mediastinal lymph-node invasion.

### Pathological Characteristics

Histopathological diagnosis was carried out on the pathological tissue sections of the patients after hematoxylin–eosin (HE) staining and immunohistochemistry, revealing a total of 58 patients with primary thyroid DLBCL. B cells were labeled with antibodies to CD20 and CD79a in all patients, BCL-2 expression in 26 patients, BCL-6 expression in 37 patients, CD10 expression in 21 patients, MYC expression in 28 patients, Myc/Bcl-2 protein co-expression in 17 patients, multiple myeloma oncogene 1 (MUM1) expression in 32 patients, and PAX5 expression in 48 patients. A proliferation index of Ki-67 ≤70% was found in 36 patients, and Ki-67 >70% in 22 patients. 21 patients had germinal center B-cell-like (GCB) DLBCL, and 37 had non-GCB DLBCL.

### Treatment and Follow-Up

Overall, 15 patients received only surgical excision (four lobectomy, eight partial lobectomy, three thyroidectomy), 31 underwent surgical biopsy, 12 underwent core needle biopsy, 18 received simple chemotherapy (CT), and 25 received chemotherapy combined with radiotherapy (RT). The radiotherapy dose ranged from 30 to 40 Gy with a median value of 36 Gy. While 26 patients were treated with rituximab, 32 did not receive the treatment. Regarding chemotherapy regimens, 17 patients received cyclophosphamide, doxorubicin, vincristine, and prednisone (CHOP) chemotherapy, whereas 26 received rituximab plus cyclophosphamide, doxorubicin, vincristine, and prednisone (R+CHOP) chemotherapy. The follow-up time was 6–120 months, with a median value of 60 months. All 58 patients had a follow-up rate of 100%. In the 58 patients with primary thyroid DLBCL, the 5-year OS and 5-year PFS were 73% (95% confidence interval (CI), 68–96%, and 61% (95% CI, 45–69%), respectively. Forty-eight patients did not show disease recurrence, whereas 10 did. Among the 10 patients with relapse, two had ipsilateral thyroid involvement, two had lung involvement, one had neck lymph-node involvement, two had brain involvement, two had bone involvement, and one had liver involvement. All the patients who experienced relapse received chemotherapy, including GEMOX (gemcitabine and oxaliplatin), MINE (mesna, ifosfamide, mitoxantrone, etoposide), and ESHAP (etoposide, methylprednisolone, cisplatin and cytarabine). Additionally, two patients also received radiotherapy, and one patient received intrathecal chemotherapy for intracranial involvement, whereas five died of progressive disease.

### Correlation Analysis of Clinical Characteristics and Treatment Methods With 5-Year PFS and OS Rates

Univariate analysis was performed for patients' age, sex, tumor size, stage, treatment modalities, B symptoms, serum LDH level, IPI score, administration of rituximab, BCL-2 protein expression level, MYC protein expression level, Myc/Bcl-2 protein co-expression, Hans classification, Ki-67, and presence of Hashimoto's thyroiditis, as listed in Table 1. The results showed that IPI score, treatment modalities, Hans classification, administration of rituximab, and Ki-67 and Myc/Bcl-2 protein co-expression were significantly associated with prognosis (*P* < 0.05), while patient age, sex, tumor size, B symptoms, Hashimoto's thyroiditis existence, BCL-2 expression level, MYC expression level, serum LDH level, and tumor stage were not significantly associated with prognosis (*P* > 0.05; Table 1).

**Table 1:** Univariate analysis affecting OS and PFS of patients with primary thyroid DLBCL.

**Clinicopathological parameters**	***n***	**5-year OS (%)**	**χ^2^**	***P***	**5-year PFS (%)**	**χ^2^**	***P***
Age, years			0.58	>0.05		1.37	>0.05
≤60	20	78.3			64.2		
>60	38	73.8			59.5		
Sex			0.93	>0.05		1.23	>0.05
Male	15	79.1			63.5		
Female	43	71.8			58.8		
Stage			1.08	>0.05		1.32	>0.05
I E	28	78.6			65		
II E	30	73.2			57.8		
IPI score			9.16	<0.05		7.32	<0.05
0–1	31	80.6			68.9		
2–3	27	51.1			47.3		
Treatments			8.97	<0.05		9.06	<0.05
Surgical excision	15	35.3			31.6		
CT	18	60.9			53.9		
CT+RT	25	81.2			77.8		
Rituximab			10.6	<0.01		9.56	<0.01
Yes	26	78.6			68.3		
No	32	50.2			47.5		
Hans classification			12.7	<0.01		11.2	<0.01
GCB	21	79.2			69.4		
Non-GCB	37	50.7			48.7		
Ki-67			11.7	<0.01		9.48	<0.01
>70%	22	57.3			46.4		
≤70%	36	78.7			65.3		
Bcl-2			1.62	>0.05		1.31	>0.05
Positive	26	71.2			57.1		
Negative	32	79.1			64.6		
Myc			1.65	>0.05		1.52	>0.05
Positive	28	73.9			58.2		
Negative	30	76.4			63.4		
Myc/Bcl-2 protein co-expression			14.2	<0.01		12.3	<0.01
Yes	17	48.9			47.3		
No	41	82.3			68.4		
LDH level			1.37	>0.05		1.33	>0.05
Normal	34	77.8			63.9		
Elevated	24	74.1			59.4		
B symptom			1.51	>0.05		1.61	>0.05
Yes	18	70.9			60.6		
No	40	78.3			63.5		
Hashimoto's thyroiditis			1.22	>0.05		1.28	>0.05
Yes	22	76.9			57.9		
No	36	72.3			64.1		
Tumor size (cm)			1.68	>0.05		1.57	>0.05
≤5	30	79.4			65.7		
>5	28	70.8			56.4		0

*n, case number; LDH, lactate dehydrogenase; IPI, international prognostic index; CT, chemotherapy; RT, radiotherapy*.

**Figure 1:**
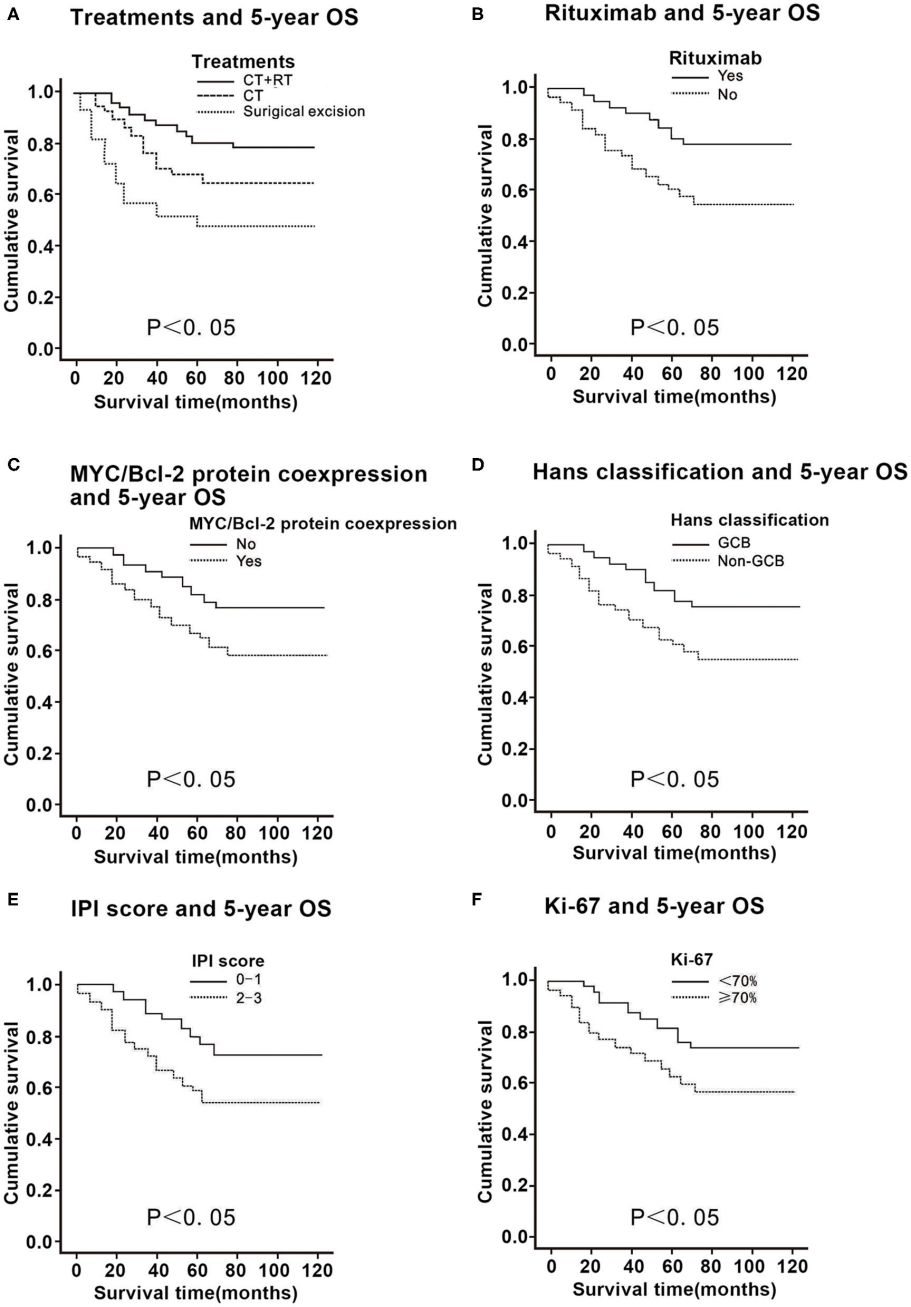
Correlation of the 5-year overall survival (OS) with the clinicopathological features in 58 patients with primary thyroid diffuse large B cell lymphoma. **(A)** Comparison of the 5-year OS in patients with primary thyroid DLBCL according to treatments. **(B)** Comparison of the 5-year OS in patients with primary thyroid DLBCL according to rituximab. **(C)** Comparison of the 5-year OS in patients with primary thyroid DLBCL according to Myc/Bcl-2 protein co-expression. **(D)** Comparison of the 5-year OS in patients with primary thyroid DLBCL according to Hans classification. **(E)** Comparison of the 5-year OS in patients with primary thyroid DLBCL according to the international prognostic index (IPI) score. **(F)** Comparison of the 5-year OS in patients with primary thyroid DLBCL according to Ki-67 expression.

**Figure 2:**
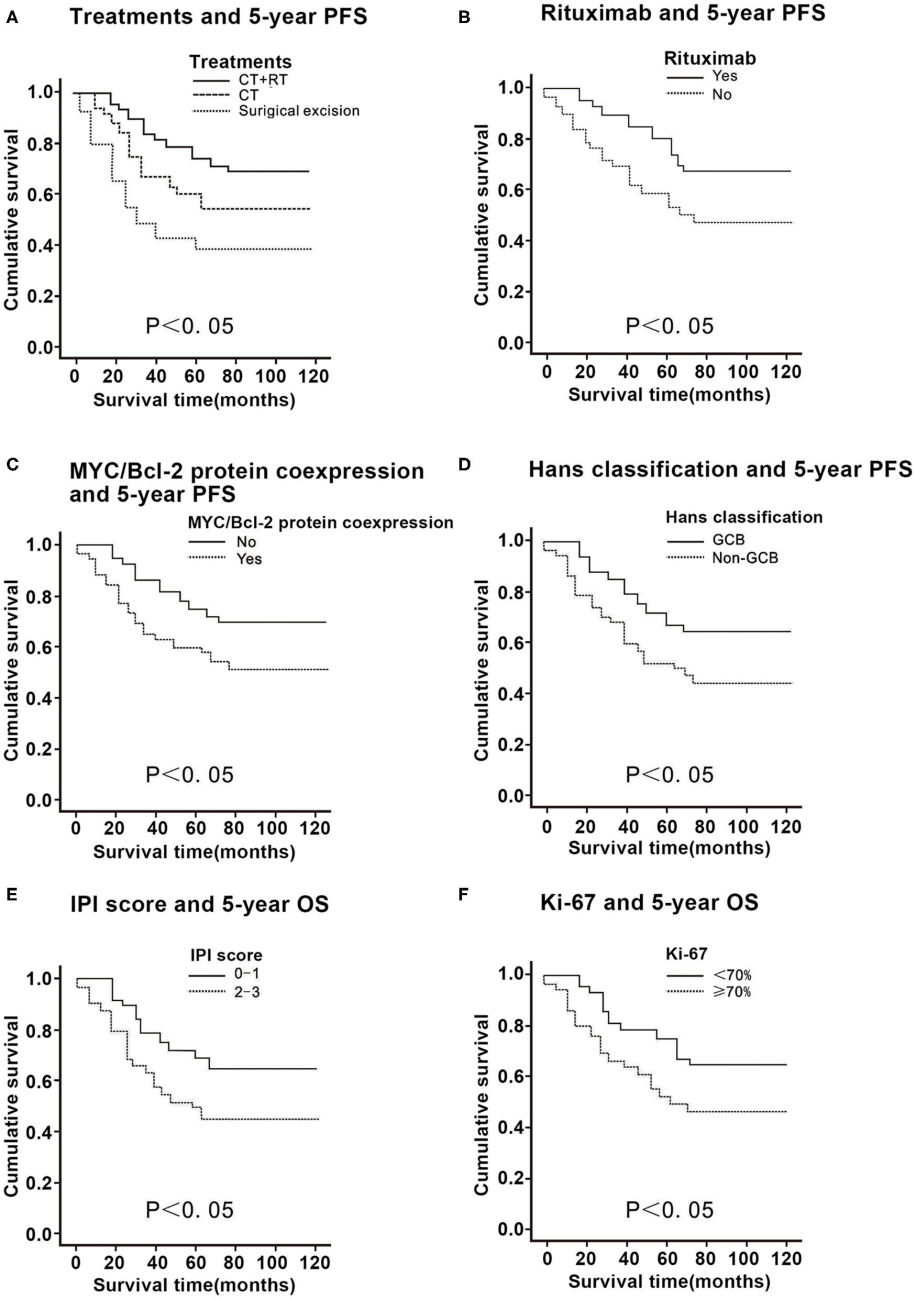
Correlation of the 5-year progression-free survival (PFS) with the clinicopathological features in 58 patients with primary thyroid diffuse large B cell lymphoma. **(A)** Comparison of the 5-year PFS in patients with primary thyroid DLBCL according to treatments. **(B)** Comparison of the 5-year PFS in patients with primary thyroid DLBCL according to rituximab. **(C)** Comparison of the 5-year PFS in patients with primary thyroid DLBCL according to Myc/Bcl-2 protein co-expression. **(D)** Comparison of the 5-year PFS in patients with primary thyroid DLBCL according to Hans classification. **(E)** Comparison of the 5-year PFS in patients with primary thyroid DLBCL according to the international prognostic index (IPI) score. **(F)** Comparison of the 5-year PFS in patients with primary thyroid DLBCL according to Ki-67 expression.

For treatment modalities, first, stratification based on baseline demographic, and clinicopathological characteristics was performed for the patients treated with different treatment modalities; the results showed that the demographic and clinicopathological characteristics were generally well-balanced among different treatment groups at baseline (Table 2). Then, survival analysis revealed that the 5-year OS and PFS were 81.2 and 77.8%, respectively, for patients with CT plus RT, 60.9 and 53.9% for patients with CT alone, and 35.3 and 31.6% for patients that underwent surgical excision (*P* < 0.05; Figures 1A, 2A), thus indicating that the patients that received CT with RT had better survival than CT or surgical excision alone and that surgical excision alone did not have any advantage over CT or CT plus RT.

**Table 2:** The baseline characteristics of the patients treated with different treatment modalities.

	**Treatment modalities**					
	**Surgical excision (*****n*** **= 15)**		**CT** **(*****n*** **= 18)**		**CT+RT** **(*****n*** **= 25)**	
**Characteristics**	***n***	**%**	***n***	**%**	***n***	**%**
Age, years						
≤60	5	33.3	7	38.9	8	32.0
>60	10	66.7	11	61.1	17	68.0
Sex						
Male	4	26.7	5	27.8	6	24.0
Female	11	73.3	13	73.2	19	76.0
Stage						
I E	7	46.7	9	50.0	12	48.0
II E	8	53.3	9	50.0	13	52.0
IPI score						
0–1	8	53.3	9	50.0	14	56.0
2–3	7	46.7	9	50.0	11	44.0
Hans classification						
GCB	5	33.3	7	38.9	9	36.0
Non-GCB	10	66.7	11	61.1	16	64.0
Ki-67						
>70%	6	40.0	7	38.9	9	36.0
≤70%	9	60.0	11	61.1	16	64.0
Bcl-2						
Positive	7	46.7	8	44.4	11	44.0
Negative	8	53.3	10	55.6	14	56.0
Myc						
Positive	7	46.7	9	50.0	12	48.0
Negative	8	53.3	9	50.0	13	52.0
Myc/Bcl-2 protein co-expression						
Yes	5	33.3	5	27.8	7	28.0
No	10	66.7	13	72.2	18	72.0
LDH level						
Normal	9	60.0	10	55.6	15	60.0
Elevated	6	40.0	8	44.4	10	40.0
B symptom						
Yes	5	33.3	5	27.8	8	32.0
No	10	66.7	13	72.2	17	68.0
Hashimoto's thyroiditis						
Yes	6	40.0	7	38.9	9	36.0
No	9	60.0	11	61.1	16	64.0
Tumor size (cm)						
≤5	8	53.3	9	50.0	13	52.0
>5	7	46.7	9	50.0	12	48.0

*n, case number; LDH, lactate dehydrogenase; IPI, international prognostic index; CT, chemotherapy; RT, radiotherapy*.

For patients who received rituximab, the 5-year OS and PFS were 78.6 and 68.3%, while for those that did not receive rituximab, the values were 50.2 and 47.5%, respectively (*P* < 0.01; Figures 1B, 2B), thus suggesting that rituximab could improve the survival rate of patients with primary thyroid DLBCL.

In patients with primary thyroid DLBCL with a positive Myc who received immunohistochemistry, the 5-year OS was 73.9% and 5-year PFS was 58.2%. In patients with primary thyroid DLBCL with a negative Myc treated by immunohistochemistry, the 5-year OS was 76.4% and the 5-year PFS was 63.4% (*P* > 0.05; Table 1). In patients with primary thyroid DLBCL with a positive Bcl-2 who underwent immunohistochemistry, the 5-year OS was 71.2% and 5-year PFS was 57.1%. In patients with primary thyroid DLBCL with a negative Bcl-2 who received immunohistochemistry, the 5-year OS was 79.2% and 5-year PFS was 64.6% (*P* > 0.05; Table 1). The 5-year OS and PFS in Myc/Bcl-2 protein co-expression patients were 48.9 and 47.3%, respectively, while those in patients without Myc/Bcl-2 protein co-expression were 82.3 and 68.4% respectively (*P* < 0.05; Figures 1C, 2C). These findings indicate that Bcl-2 protein expression or Myc protein expression alone has no significant effect on survival, but Myc/Bcl-2 protein co-expression is negatively associated with prognosis, i.e., the patients with Myc/BCL-2 protein co-expression had a worse prognosis.

For Hans classification, the 5-year OS and 5-year PFS were 79.2 and 69.4% for the patients with GCB DLBCL and those of the patients with non-GCB DLBCL were 50.7 and 48.7%, respectively (*P* < 0.05; Figures 1D, 2D), indicating that non-GCB subtype was significantly associated with poor prognosis.

For patients with an IPI score of 0–1, the 5-year OS was 80.6% and the 5-year PFS was 68.9%, while for patients with an IPI score of 2–3, the 5-year OS and the 5-year PFS were 51.1 and 47.3%, respectively (*P* < 0.05). Thus, the higher the IPI score, the worse the prognosis (Figures 1E, 2E).

The 5-year OS of patients with a proliferation index of Ki-67 ≤70% was 78.7%, and the 5-year PFS was 65.3%; the 5-year OS of patients with a proliferation index of Ki-67 >70% was 57.3%, and the 5-year PFS was 46.4% (*P* < 0.05), indicating that patients with a proliferation index of Ki-67 >70% had a worse prognosis (Figures 1F, 2F).

Furthermore, multivariate risk regression analysis using the Cox model revealed that Myc/Bcl-2 protein co-expression, treatment modalities, and rituximab were independent prognostic factors for patients with primary thyroid DLBCL (*P* < 0.001; Table 3).

**Table 3:** Multivariate analysis and regression analysis of COX model affecting the prognosis of patients with primary thyroid DLBCL.

**Clinicopathological parameters**	**β**	**SE**	**OR**	**χ2**	**P**	**95% CI**
CT+RT	2.19	0.58	1.85	12.01	0.001^*^	0.261~0.662
IPI	−1.04	0.85	0.37	1.59	0.312	0.174~2.791
Hans classification	0.46	0.56	0.45	1.47	0.347	0.374~1.034
Rituximab	−1.07	0.36	1.37	9.06	0.001^*^	0.17~0.79
Myc/Bcl-2 protein co-expression	2.21	0.59	1.65	11.08	0.005^*^	2.71~3.46
Ki-67	0.39	0.67	0.19	0.29	0.762	0.348~3.745

* *P < 0.05 was statistically significant. SE, standard error; OR, odds ratio; CI, confidence interval; CT, chemotherapy; RT, radiotherapy*.

## Discussion

PTL is a rare neoplasm of the thyroid gland, with the most common type of PTL being DLBCL. This was a larger study that evaluated the association of clinicopathological characteristics and treatments with a survival rate in patients with primary thyroid DLBCL. With 58 cases in the final analysis, some important findings were revealed for the first time in our study.

Optimal treatment methods for primary thyroid DLBCL have not been established (2, 3). The role of surgery in PTL has changed (7, 8); although it is usually used as diagnostic biopsies now, some patients with PTL were reported to have undergone surgical excision because of various reasons (3) In our study, 15 patients underwent surgical excision; however, even with lesion resection, these patients had no significant prognostic advantage. A study from the Mayo Clinic showed that surgical excision did not have any advantage over the simply performed surgical biopsy for diagnosis. Patients who underwent surgical biopsy and chemotherapy had better prognosis than those with surgical excision followed by adjuvant chemotherapy (9). Therefore, chemotherapy and RT have replaced surgery as the main treatment measure (8). Mian et al. (10) reported that patients who underwent chemotherapy followed by radiotherapy had a longer PFS than those who received single therapy. Doria et al. (11) reported that the recurrence rate was 7.7% with combined therapy of radiotherapy and chemotherapy for thyroid primary DLBCL, 37.1% with radiotherapy alone, and 43% with chemotherapy alone. Matsuzuka et al. (12) reported the outcome of chemotherapy plus RT in patients with aggressive PTL, in which the survival rate at 8 years was 100%. Onal et al. (13) described the treatment effect of 60 patients with aggressive PTL; among those cases, there was a remarkable improvement in survival rate and local control with chemotherapy plus RT over RT or chemotherapy alone. The findings from our study also demonstrated that patients with primary thyroid DLBCL receiving CT plus RT had better prognosis, supporting the role for combined therapy of chemotherapy plus radiotherapy in those with aggressive histologic subtypes. Targeted CD20 therapy is widely being used for DLBCL with CD20 positive. CD20 is positive in primary thyroid DLBCL, and in the present study, CD20 is positive in all 58 cases with primary thyroid DLBCL; therefore, immunochemotherapy including rituximab might be a good treatment. In this study, chemotherapy combined with rituximab confers additional benefits to the patients with primary thyroid DLBCL. Consistent with our result, Vardell et al. (14) reported that CHOP plus rituximab showed better results for the management of primary thyroid DLBCL. Dralle et al. (15) suggested that rituximab could improve survival of patients with primary thyroid DLBCL. Watanabe et al. (16) showed that the inclusion of rituximab to combination therapy was effective for elderly patients with primary thyroid DLBCL. These findings suggested that chemotherapy including rituximab in combination with supplementary radiotherapy may be optimal treatment modality for patients with primary thyroid DLBCL.

Furthermore, this study also investigated the association between pathological features and prognosis in patients with primary thyroid DLBCL. Alterations in Myc gene and Bcl-2 gene, which are main regulators of cellular apoptosis and proliferation, may be related to the pathogenesis of DLBCL (17, 18). Some DLBCL also co-express high levels of Bcl-2 protein and Myc protein, which may be a key factor of pathogenesis in this disease. In DLBCLs, Myc overexpression, Bcl-2 overexpression, and Myc/Bcl-2 protein co-expression were detected by IHC staining in 29–47% and 50% and 20–35% of the cells, respectively, (19, 20). This study showed 44.8% were positive for Bcl-2, 48.3% were positive for Myc, and 29.3% were double positive for Myc and Bcl-2 protein expression. Whether Myc or Bcl-2 expression in IHC singly predicts survival outcome is controversial, with some studies showing an inferior outcome (19, 20) and others showing no association with outcome (21). However, Myc/Bcl-2 protein co-expression, the so-called double-expresser phenotype, is considered a useful prognostic tool for predicting survival outcome (19, 20). Despite that several studies showed an association of IHC-determined Myc and Bcl-2 expression with inferior outcomes in nodal DLBCL (20), there have been very few reports of Myc/Bcl-2 co-expression in primary thyroid DLBCL. Consistent with results from prior studies, this study showed that Bcl-2 or Myc protein expression alone had no significant effect on survival, while patients with primary thyroid DLBCL with Myc/Bcl-2 protein co-expression had poor clinical outcomes, with <30% rate of 5-year OS and PFS.

The positive cell rate of Ki-67 expression is referred to as the proliferation activity of cells. The prognostic value of Ki-67 expression in patients with DLBCL remains controversial. Some studies found a significantly negative relationship of prognosis with high Ki-67 expression (22); other studies showed no effect on prognosis (22). In this study, for patients with high Ki-67 expression (>70%), the 5-year OS and PFS were much lower than those in patients with low Ki-67 expression (≤70%). Thus, we inferred that Ki-67 expression status had a prognostic value to survival. Patients with high Ki-67 expression also seem to have a shorter survival. Probable explanations for the inter-report variations are differences in study populations and cutoff value of Ki-67 (22, 23).

Recent studies have shown that patients with DLBCL may be divided into two types by the Hans classification, such as GCB type and non-GCB type; previous studies showed that the majority of patients with primary extra-nodal DLBCL are of the non-GCB (24). Furthermore, the GCB type is correlated with a better prognosis than the non-GCB type (25). Consistent with prior studies, in the present study, 63.8% patients with primary thyroid DLBCL were of non-GCB type, accounting for a majority of these cases. 5-year OS and PFS in patients with GCB type were significantly higher than were patients with the non-GCB type.

IPI has been reported to stratify patients with aggressive lymphoma into distinct groups; IPI remains one of the main indices for assessing prognosis in DLBCL, even in the current era of immunochemotherapy including rituximab (26). This study also confirmed this finding; the results showed an association of high IPI scores with poor prognosis in primary thyroid DLBCL.

In our study, 26.5% of patients presented with B symptoms, 41.4% of patients had high serum levels of LDH, 51.7% of patients had stage IE lymphoma, and there were 43.8% of patients with tumor size >5 cm. Previous studies had shown that B symptoms, high serum LDH levels, bulky tumor, and advanced stage were positively correlated with poor prognosis in nodal DLBCL (3, 27, 28). However, in our study, B symptoms, serum levels of LDH, tumor size, and clinical stage were not associated with prognosis. We infer that primary thyroid DLBCL was different from nodal DLBCL in biological activity.

Hashimoto's thyroiditis is an established risk factor for PTL often accompanying PTL and a potential precursor that confers a 67- to 80-fold increase in risk (2, 29). In our study, 39% of the patients showed features of Hashimoto's thyroiditis; however, its presence was not significantly associated with 5-year OS and PFS.

In summary, this study suggested that surgery did not improve the prognosis of patients with primary thyroid DLBCL; chemotherapy including rituximab in combination with supplementary radiotherapy may be an optimal treatment modality for patients with primary thyroid DLBCL. Myc/Bcl-2 protein co-expression, rituximab, and treatment modalities were found to be independent factors of prognosis in patients with primary thyroid DLBCL. Due to the rarity of this disease, some multicenter, and prospective trials are required to guide the clinical treatment of primary thyroid DLBCL in future.

## Significance

This study revealed that patients with primary thyroid diffuse large B cell lymphoma who received combination immunochemotherapy with radiotherapy had a better prognosis and Myc/Bcl-2 protein co-expression, treatment modalities, and rituximab were independent prognostic factors.

## Data Availability Statement

The original contributions presented in the study are included in the article/supplementary material, further inquiries can be directed to the corresponding author/s.

## Ethics Statement

Written informed consent was obtained from the individual(s) for the publication of any potentially identifiable images or data included in this article.

## Author Contributions

JY, PF, and PY designed this study. JY, PY, HL, and XW performed the search and collected the data. PF, WW, HW, and PY rechecked the data. JY, PF, and PY performed the analysis and wrote the manuscript. All authors approved the final version of the manuscript.

## Conflict of Interest

The authors declare that the research was conducted in the absence of any commercial or financial relationships that could be construed as a potential conflict of interest.
